# SpatialCell AI achieves reference-free single-cell resolution from spot-based spatial transcriptomics through morphology-guided enhancement

**DOI:** 10.1038/s41598-026-55246-w

**Published:** 2026-06-16

**Authors:** Abdalla Elbialy

**Affiliations:** SpatialCell AI, LLC, Oak Lawn, IL USA

**Keywords:** Spatial transcriptomics, Single-cell analysis, Cell segmentation, Morphological features, Computational biology, Validation framework, Biological techniques, Cancer, Cell biology, Computational biology and bioinformatics

## Abstract

Spatial transcriptomics platforms such as 10x Visium capture whole-transcriptome expression but at spot-level resolution where each measurement aggregates multiple cells. Existing computational deconvolution methods require external scRNA-seq references, and the growing set of morphology-guided methods (iStar, SpaHDmap, GHIST, Thor, PRTS) each requires per-dataset model training or paired subcellular spatial data. Here we present SpatialCell AI, the first framework to combine training-free operation, reference-free expression integration, and per-cell output granularity for spatial transcriptomics. We validated SpatialCell AI on a matched colorectal cancer sample analyzed across Visium (55 μm), Visium HD (8 μm and 16 μm), and Xenium (single-cell ground truth). On Visium HD 8 μm input, SpatialCell AI achieves an expression correlation of *r* = 0.791 against the Xenium reference — the highest of any method tested on this sample. Across six distribution-based validation metrics on the matched 408-gene panel, the SpatialCell AI HD variants lead on the majority of metrics, with no competing method winning on any. A strict matched-region head-to-head comparison reveals a clear architectural distinction: training-free integration improves monotonically as input bins approach single-cell scale, while the trained morphology-guided methods iStar and SpaHDmap track the raw baseline on every platform. SpatialCell AI achieves its strongest performance on Visium HD inputs, where it substantially outperforms both raw baselines and competing morphology-guided methods while transforming spot-level data into individual cell records with spatial coordinates and per-cell expression — an output no raw spot measurement can provide.

**Supplementary Information:**

The online version contains supplementary material available at https://doi.org/10.1038/s41598-026-55246-w.

## Introduction

Spatial transcriptomics, designated “Method of the Year 2020,” has emerged as an important technology enabling simultaneous measurement of gene expression and spatial localization within intact tissues^1^. This capability provides valuable insights into tissue architecture, cellular microenvironments, and spatially organized biological processes that are lost in traditional dissociation-based single-cell approaches^2, 3^.

Current spatial transcriptomics technologies broadly fall into two categories^4^: (1) image-based methods (MERFISH, seqFISH, osmFISH) that provide single-cell or subcellular resolution but are limited to hundreds of genes, and (2) sequencing-based methods (10x Visium, Spatial Transcriptomics, Slide-seq^5^) that capture whole-transcriptome profiles but at spot-level resolution where each measurement represents multiple cells^6, 7^. This fundamental trade-off between spatial resolution and transcriptomic breadth represents a critical bottleneck in the field. Most widely-used platforms, including the 10x Visium system, capture expression from multiple cells per spot (55 μm diameter), limiting biological interpretation. While recent technologies like Stereo-seq and Xenium achieve higher resolution, they come with significant limitations in cost, tissue coverage, or gene panel size^8^.

### The cell type deconvolution challenge

The predominant approach to address spot-level limitations has been computational cell type deconvolution — inferring the cellular composition and proportions within each spot using external single-cell RNA sequencing (scRNA-seq) reference datasets^9, 10^. Methods such as RCTD^10^, cell2location^9^, and SpatialDWLS^11^ have shown success in estimating cell type proportions. Three comprehensive benchmarking studies have systematically evaluated this landscape: Li et al. (2023) benchmarked 18 methods across 50 datasets^12^; Chen et al. (2022) compared 10 methods using real datasets^13^; and Li et al. (2022) evaluated 16 methods for transcript distribution prediction^14^. Collectively, these studies evaluated 28 + distinct computational approaches encompassing probabilistic methods, non-negative matrix factorization, graph-based approaches, optimal transport methods, and deep learning frameworks.

Despite this methodological diversity, three shared limitations constrain the utility of these approaches. First, reference dependency: 25 of 28 methods require external scRNA-seq datasets from matched tissues, creating a barrier for novel tissue types or rare diseases^12, 13, 14^. Second, platform effects: systematic differences between reference and target data cause accuracy drops of 30–50% in real-world applications^12^. Third, sparse-data sensitivity: spatial transcriptomics data inherently have lower gene detection rates than traditional scRNA-seq^15^, and the requirement for cell type matching means methods fail when encountering novel or patient-specific cell states not represented in reference databases^16^. While a small but growing number of methods do integrate histological image features with spatial transcriptomics (see *Morphology-guided methods and the position of SpatialCell AI*), the majority of computational deconvolution approaches operate purely at the level of spot expression without leveraging tissue morphology.

### Morphology-guided methods and the position of SpatialCell AI

A growing family of methods integrates histology images with spot-based spatial transcriptomics to enhance effective resolution. **iStar**^17^ trains a hierarchical vision transformer on paired image–transcriptome tiles to predict super-resolved gene expression at the superpixel level. **SpaHDmap**^18^ fits a multimodal neural network per dataset to learn joint image–transcriptome embeddings and outputs interpretable metagene maps at the pixel scale. **GHIST**^19^ couples histology features with paired subcellular spatial transcriptomics (e.g. Xenium, CosMx) to predict cell-level expression from H&E patches. **Thor **uses a graph-attention model on H&E features to refine Visium expression. **PRTS** segments nuclei from H&E and uses a learned encoder to predict per-cell expression vectors directly from image patches. Each of these methods requires either per-dataset model training, paired subcellular spatial data, or both (Table 1).

SpatialCell AI occupies a previously unfilled position in this landscape. Unlike iStar and SpaHDmap, it requires no model training; unlike GHIST and PRTS, it requires no paired subcellular spatial data and no learned image encoder; unlike the reference-based deconvolvers (RCTD, cell2location, Tangram, Stereoscope, DestVI), it requires no scRNA-seq reference. The expression-integration step requires no training, no reference data, and no per-dataset model fitting (see* Methods, Step 4*). The single learned component anywhere in the pipeline is a pretrained convolutional neural network for nuclear segmentation on H&E and fluorescent images. SpatialCell AI is the first framework to combine (i) pretrained nuclear segmentation, (ii) training-free expression integration, and (iii) per-cell output granularity, in a pipeline that can be applied to any tissue and any spot-based platform without external data.

An open question, which this manuscript addresses, is whether training-free morphology-guided integration can match or exceed the performance of trained methods — and under what input-resolution conditions. The validation in (see *Results*) tests this directly by comparing SpatialCell AI against iStar and SpaHDmap across three Visium input platforms (55 μm, HD 16 μm, HD 8 μm) on the same tissue sample with matched preprocessing.

**Table 1:** Method comparison matrix.

Method	Segmentation source	Per-dataset training?	Reference data required?	Output granularity
**SpatialCell AI**	Pretrained CNN for nuclear segmentation on H&E and fluorescent images	None	None for integration	Individual segmented cells
iStar	None (image tiles)	Hierarchical ViT trained per dataset	Paired ST tiles	Superpixels (≈ µm scale)
SpaHDmap	None (image patches)	Multimodal NN trained per dataset	Paired ST patches	Pixel-level metagene maps
GHIST	Built into model	End-to-end trained	**Paired Xenium/CosMx** for training	Cells (predicted, not segmented)
Thor	None (image features)	Graph-attention model fit	Paired ST	Refined spot expression
PRTS	Segmentation network	Encoder trained	Paired single-cell spatial	Cells (predicted)
RCTD	None	Per-dataset cell-type fit	scRNA-seq reference	Cell-type fractions per spot
cell2location	None	Bayesian model fit	scRNA-seq reference	Cell-type fractions per spot
Tangram	None	Joint embedding optimization	scRNA-seq or Xenium reference	Mapped cells from reference
Stereoscope	None	Probabilistic model fit	scRNA-seq reference	Cell-type fractions per spot
DestVI	None	VAE fit	scRNA-seq reference	Cell-type fractions per spot

Side-by-side comparison of SpatialCell AI against morphology-guided methods (iStar, SpaHDmap, GHIST, Thor, PRTS) and reference-based deconvolution methods (RCTD, cell2location, Tangram, Stereoscope, DestVI). SpatialCell AI is the only method that requires no per-dataset training, no external reference data, and produces per-cell output.

## Methods

### Pipeline overview

SpatialCell AI is a training-free pipeline that converts a histological image and a spot-based spatial transcriptomics expression matrix into a per-cell expression matrix. The pipeline integrates two complementary data streams: (i) morphological features extracted from H&E-based nuclear segmentation (cell shape, area, density, and spatial organization) and (ii) gene expression from spot-based spatial transcriptomics. In the Basic processing mode, these streams are combined through spatial proximity; in the Advanced mode, cell-type-specific marker genes are additionally incorporated into the expression integration, enabling biologically guided assignment informed by known marker signatures for cell types such as epithelial, immune, and stromal populations. No single-cell reference is used during expression integration. The pipeline consumes raw outputs from 10x Visium or Visium HD (binned outputs at the desired resolution: 8 μm, or 16 μm) and emits a per-cell expression matrix containing one row per segmented cell with spatial coordinates and integrated expression.

Step 1 — Histology preprocessing and nuclear segmentation

The slide image — H&E or immunofluorescent (IF) staining from a Visium or Visium HD experiment — is preprocessed and passed to a pretrained convolutional neural network for nuclear segmentation. It produces a polygon-based mask for each detected nucleus; the centroid of each polygon is taken as the cell location, and the polygon itself is retained for downstream morphological feature extraction.

Step 2 — Morphological feature extraction and cell identification

For each segmented nucleus, a comprehensive set of morphological features is extracted from the polygon mask, including spatial coordinates, size descriptors (area, perimeter), shape descriptors (eccentricity, solidity, axis ratios), texture features, and local density measures. These morphological features are then used to identify and classify cells based on their physical characteristics — different cell types exhibit distinct morphological signatures in stained tissue (e.g., nuclear size, shape regularity, intensity texture, and spatial density patterns), with the same feature set applied to both H&E and IF inputs. This morphology-based cell identification constitutes the first of the two complementary data streams described in the pipeline overview, and provides cell-type context that is independent of gene expression.

Step 3 — Spot-to-cell registration

Each segmented cell is paired with nearby spots based on spatial proximity. In the **Basic** mode, pairing is based on spatial proximity. In the **Advanced** mode, cell-type-specific marker genes are additionally incorporated, enabling biologically informed assignment of spot expression to cells based on both spatial proximity and known marker-gene signatures.

Step 4 — Reference-free expression integration

Each segmented cell is assigned an expression vector by integrating the expression of nearby spots using a deterministic integration step that requires **no training** and no external reference. This is the central methodological distinction from neural integration methods such as iStar, GHIST, and SpaHDmap, all of which require per-dataset model training (see *Morphology-guided methods and the position of SpatialCell AI* ).

Step 5 — Output generation and quality filtering

The integrated per-cell expression matrix undergoes standard quality control filtering^20^. The pipeline outputs data in the same standard formats as the input (H5, H5AD, CSV) — but at per-cell resolution rather than per-spot. This means the output is directly compatible with existing single-cell and spatial transcriptomics analysis workflows without format conversion, enabling seamless downstream analysis with standard tools.

### Cell-type assignment

Cell-type labels are assigned by matching each cell’s expression profile against a marker-gene database. All validation against Xenium (see *Distribution-based validation across all methods, Robustness across two additional orthogonal metrics*, and *Head-to-head benchmark against morphology-guided baselines*) operates on the per-cell expression matrix only.

### Pipeline variants used in the validation study

Four processing variants of SpatialCell AI are evaluated. The expression-integration step is identical across all four variants. The variants differ in (i) which spot input layer is consumed and (ii) whether the Advanced processing mode is used.

1. **Visium Basic** — Visium 55 μm spots as input, Basic processing mode.

2. **Visium Advanced** — Visium 55 μm spots as input, Advanced processing mode.

3. **Visium HD 8 μm** — Visium HD 8 μm bins as input.

4. **Visium HD 16 μm** — Visium HD 16 μm bins as input.

Segmentation behaviour is identical across all four variants. The validation metrics reported in *Distribution-based validation across all methods *through *Head-to-head benchmark against morphology-guided baselines *are computed on the per-cell expression matrix only; any difference in those metrics between Basic and Advanced reflects the processing mode, not a change in the underlying integration architecture.

### Hardware footprint

Compute requirements scale with input resolution. The Visium 55 μm pipeline runs on a standard workstation. The Visium HD 8 μm and 16 μm pipelines, which integrate over very large bin counts, require a high-memory server and are typically run on dedicated infrastructure.

### Choice of validation metric: per-gene mean Pearson

The primary validation metric used throughout this manuscript (see *Distribution-based validation across all methods, Head-to-head benchmark against morphology-guided baselines*, Supplementary Table S1) is the **per-gene mean Pearson correlation against Xenium ground truth**: for each of the 408 Visium ∩ Xenium panel genes, we compute the mean expression across all cells (or spots, or super-pixels) in the predicted output, then correlate the resulting gene-mean vector against the corresponding gene-mean vector from the Xenium single-cell reference.

The per-cluster fraction Pearson metric typically used in classical reference-based deconvolution benchmarks (RCTD, cell2location, Tangram, Stereoscope, DestVI) measures a different quantity: it measures how well a method recovers per-spot cell-type *fractions*, which presupposes a reference-defined cluster vocabulary and operates at spot rather than cell granularity. SpatialCell AI’s native output is per-cell expression — a gene × cell matrix — and the appropriate validation question is whether the gene-level expression distribution matches the Xenium reference. Because these methods produce fundamentally different outputs, we report each on its own native metric (per-gene Pearson for SpatialCell AI in  *Distribution-based validation across all methods, Head-to-head benchmark against morphology-guided baselines*, and Supplementary Table S1; per-cluster fraction Pearson for Tangram and cell2location in *Pseudo-spot deconvolution benchmark against reference-based methods*, Supplementary Table S2) and do not combine them on the same axes anywhere in the manuscript or supplementary figures. The per-gene Pearson values for SpatialCell AI are computed using the publicly available validation framework (https://github.com/Spatialcell/spatialcell-ai-validation/).

### Evaluation of morphology-guided baseline methods (iStar and SpaHDmap)

The same per-gene mean Pearson framework was applied to the two morphology-guided comparator methods using their publicly available releases on the matched Oliveira P2 sample. iStar^17^ was selected because it is the only morphology-guided method with publicly available pretrained weights that can be applied to new data without per-dataset training; GHIST^19^, Thor, and PRTS all require training on paired subcellular spatial data or per-dataset model fitting (Table 1). SpaHDmap^18^ is included as the second comparator because it can produce per-gene super-resolution maps from its trained metagene basis, enabling a per-gene Pearson comparison on a matched gene subset.

We ran iStar^17^ v1.0.0 under matched preprocessing across all three Visium input platforms on the Oliveira P2 sample: standard Visium 55 μm (10k × 10k crop, 740 spots), Visium HD 16 μm bins (20k × 20k crop, 106,168 bins), and Visium HD 8 μm bins (14k × 14k crop, 210,225 bins). The gene set was restricted to the 408 Visium ∩ Xenium panel genes used for the raw spot baselines and SpatialCell AI entries in Supplementary Table S1, allowing direct comparison on identical genes.

We ran SpaHDmap^18^ v0.1.5 end-to-end at the standard rank = 20 configuration on the matched crops for all three Visium platforms. Per-gene super-resolution maps were recovered by projecting each pixel’s learned embedding through the trained metagene basis. SpaHDmap’s standard pipeline auto-selects approximately 3,000 highly variable genes (HVGs) and produces no expression output for genes outside this set. Of the 408 panel genes, 246 were among SpaHDmap’s HVGs at 55 μm, 215 at HD 16 μm, and 212 at HD 8 μm. Scoring was performed on these panel ∩ HVG intersections.

SpaHDmap’s default gene selection was not overridden, because supplying a non-default gene set would place SpaHDmap in a non-standard configuration. Detection rate is not reported for SpaHDmap because its continuous pixel-level output is nonzero at essentially every pixel, yielding near-unity detection rate for all genes — uninformative as a measure of sparsity preservation.

## Results

**Fig. 1:**
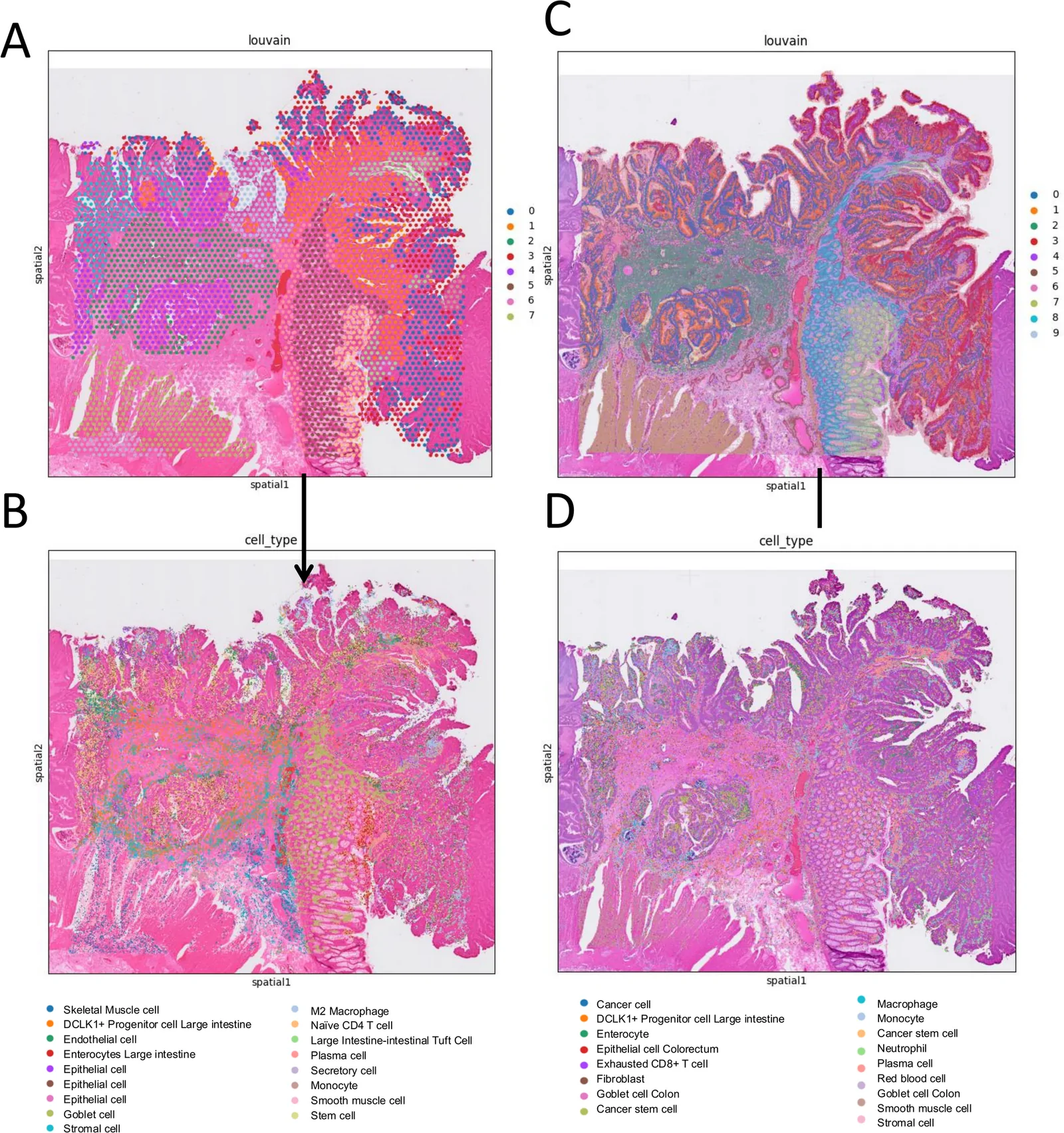
SpatialCell AI transforms spot-based spatial transcriptomics data to true single-cell resolution. Representative colorectal cancer tissue sections before and after SpatialCell AI processing, visualised by Louvain cluster colouring. **(A)** Standard Visium (55 μm) input: spot-level clusters. **(B)** Standard Visium (55 μm) output: per-cell clusters at single-cell resolution. **(C)** Visium HD (16 μm) input: bin-level clusters. **(D)** Visium HD (16 μm) output: per-cell clusters at single-cell resolution.

To demonstrate the capability of SpatialCell AI in transforming spot-based spatial transcriptomics data to single-cell resolution, we first present visual evidence of the enhancement process across different platform resolutions (Fig. 1). The standard Visium platform (55 μm spot diameter) captures expression from approximately 10–20 cells per spot, resulting in mixed cellular signals that obscure individual cell identities (Fig. 1A). Following SpatialCell AI processing, these multi-cellular spots are successfully deconvolved into individual cells with distinct cell type assignments and precise spatial coordinates (Fig. 1B). This transformation reveals fine tissue architecture and cellular interactions previously hidden by spot-level signal averaging.

Similarly, Visium HD data at 16 μm resolution, while capturing fewer cells per spot (typically 2–5 cells), still suffers from cellular signal mixing (Fig. 1C). SpatialCell AI processing of this higher-resolution input demonstrates substantial enhancement, resolving individual cells even in densely packed tissue regions (Fig. 1D). The morphology-guided approach enables accurate identification of cellular boundaries and preservation of spatial relationships, critical for understanding tissue microenvironments. These visual results establish that SpatialCell AI successfully extracts single-cell information from spot-based measurements across different input resolutions, setting the stage for rigorous quantitative validation.

**Fig. 2:**
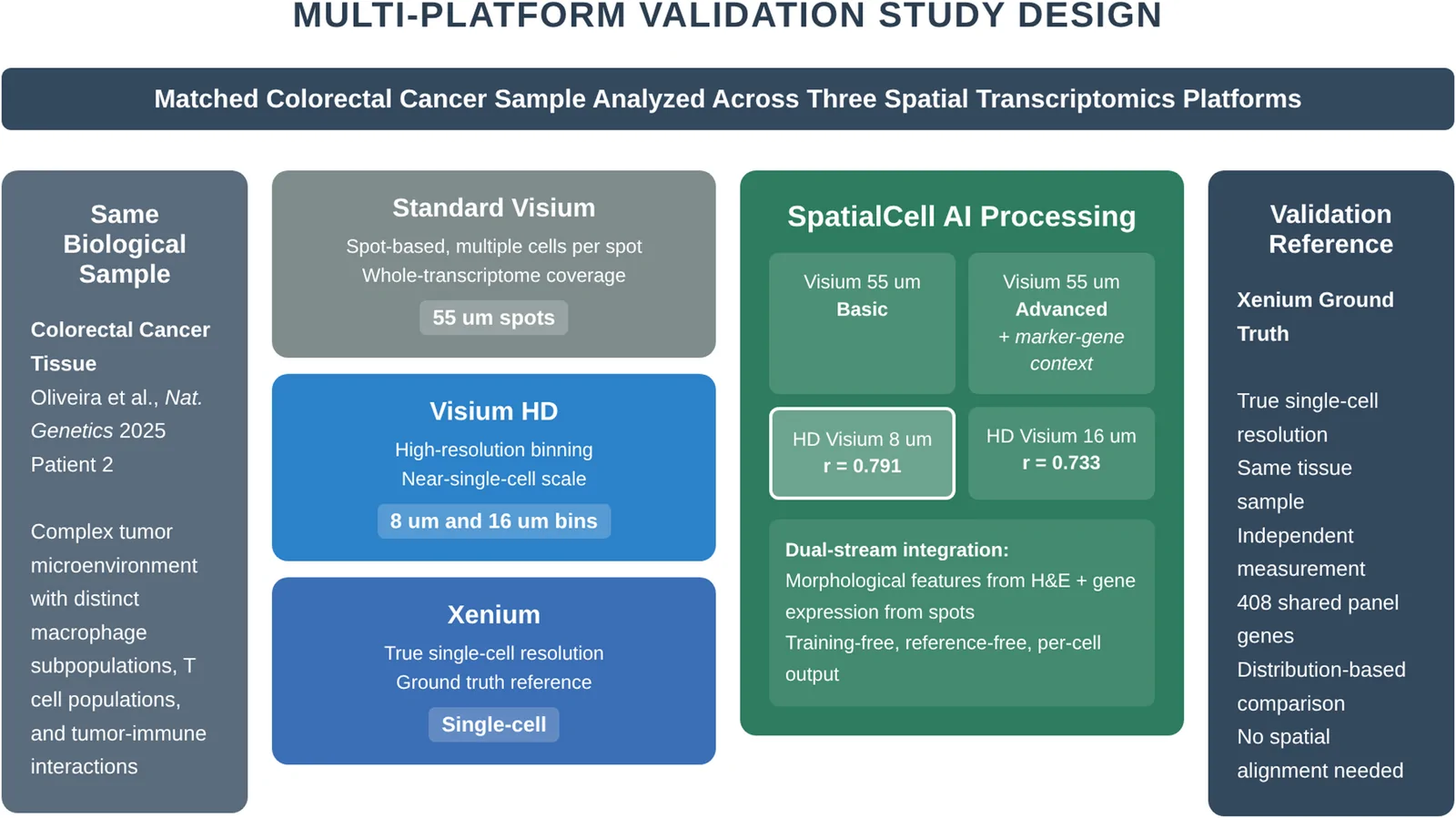
Multi-Platform Validation Study Design. Schematic of the multi-platform validation study design. The same colorectal cancer tissue sample (Oliveira et al., *Nat. Genetics* 2025, Patient 2) was analyzed across three spatial transcriptomics platforms: Standard Visium (55 μm), Visium HD (8 μm and 16 μm), and Xenium (single-cell reference). SpatialCell AI integrates morphological features from H&E segmentation with gene expression from spots, and was applied using Basic and Advanced processing modes, generating four variants (Visium Basic, Visium Advanced, Visium HD 8 μm, Visium HD 16 μm) validated against Xenium.

To rigorously validate SpatialCell AI, we utilized matched colorectal cancer tissue samples from the High-definition spatial transcriptomic profiling study by Oliveira et al. (Nature Genetics, 2025)^21^. This dataset provided a unique opportunity to validate computational enhancement using three spatial transcriptomics platforms from the same biological samples: standard Visium (55 μm spot diameter), Visium HD with two binning strategies (8 μm and 16 μm), and Xenium (single-cell resolution ground truth) (Fig. 2A). Xenium was selected as the validation reference because it provides true single-cell resolution gene expression measurements^22^ from the same tissue samples, offering an independent ground truth dataset for evaluating the accuracy of our computational single-cell enhancement approach. Unlike spot-based technologies that capture expression from multiple cells, Xenium directly measures gene expression at individual cell resolution, making it the ideal standard for validating whether SpatialCell AI can accurately reconstruct single-cell information from multi-cellular spot measurements. The colorectal cancer tumor microenvironment, with its complex cellular architecture including distinct macrophage subpopulations, T cells, and tumor cells, provided an ideal testbed for evaluating single-cell resolution enhancement across diverse cell types and spatial niches.

### Distribution-based validation across all methods

**Fig. 3:**
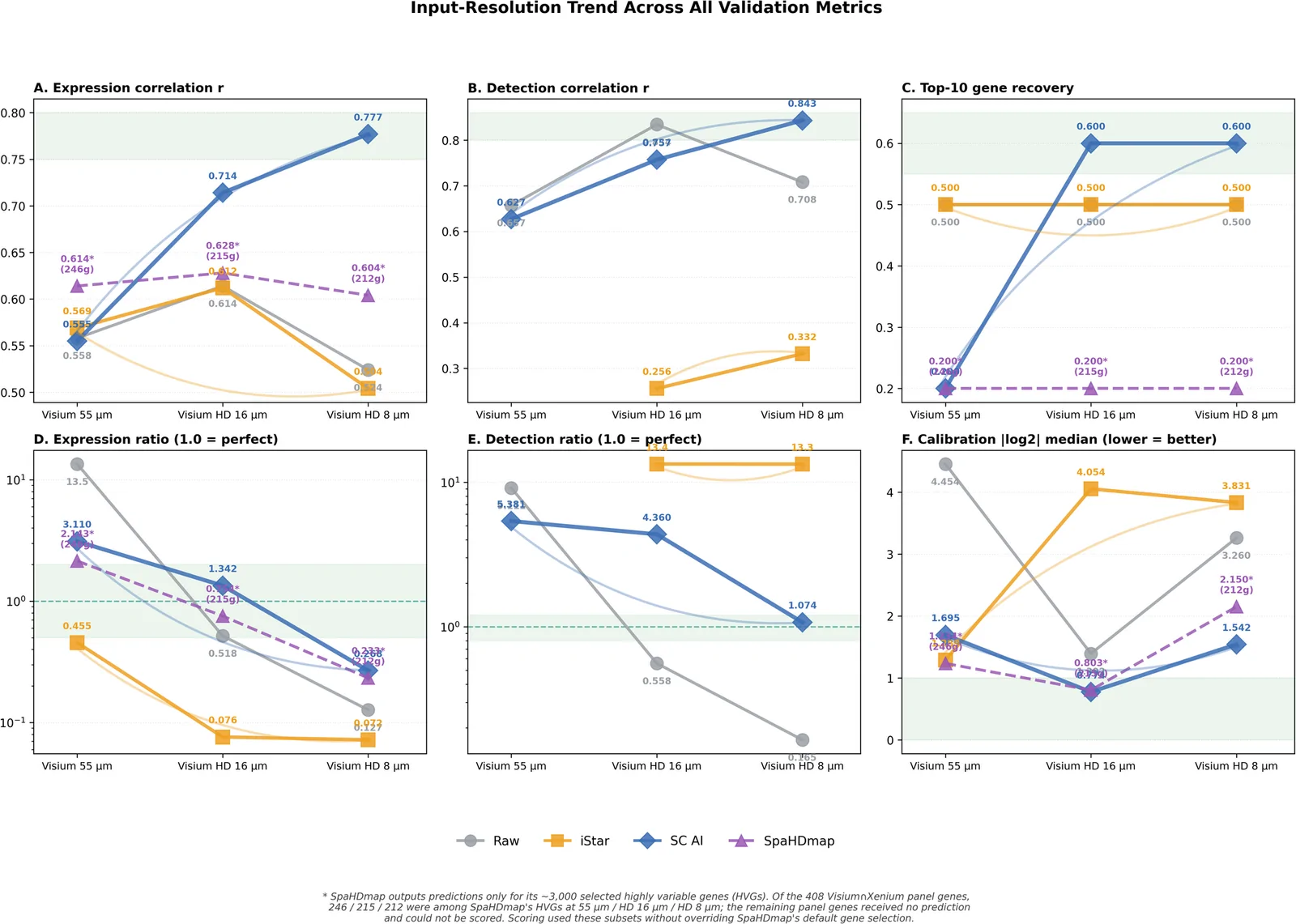
Input-Resolution Trend Across All Validation Metrics. Four methods tracked across the three Visium input platforms (55 μm → HD 16 μm → HD 8 μm) for all six validation metrics, on a strict matched-crop comparison against the Xenium single-cell reference. Genes used: 408 Visium ∩ Xenium panel for raw, iStar, and SpatialCell AI; panel ∩ HVG subset for SpaHDmap. Lines coloured by method: raw spot baseline (grey), iStar (orange), SpaHDmap (purple), SpatialCell AI (blue). Green bands mark the performance zone for each metric.

**Fig. 4:**
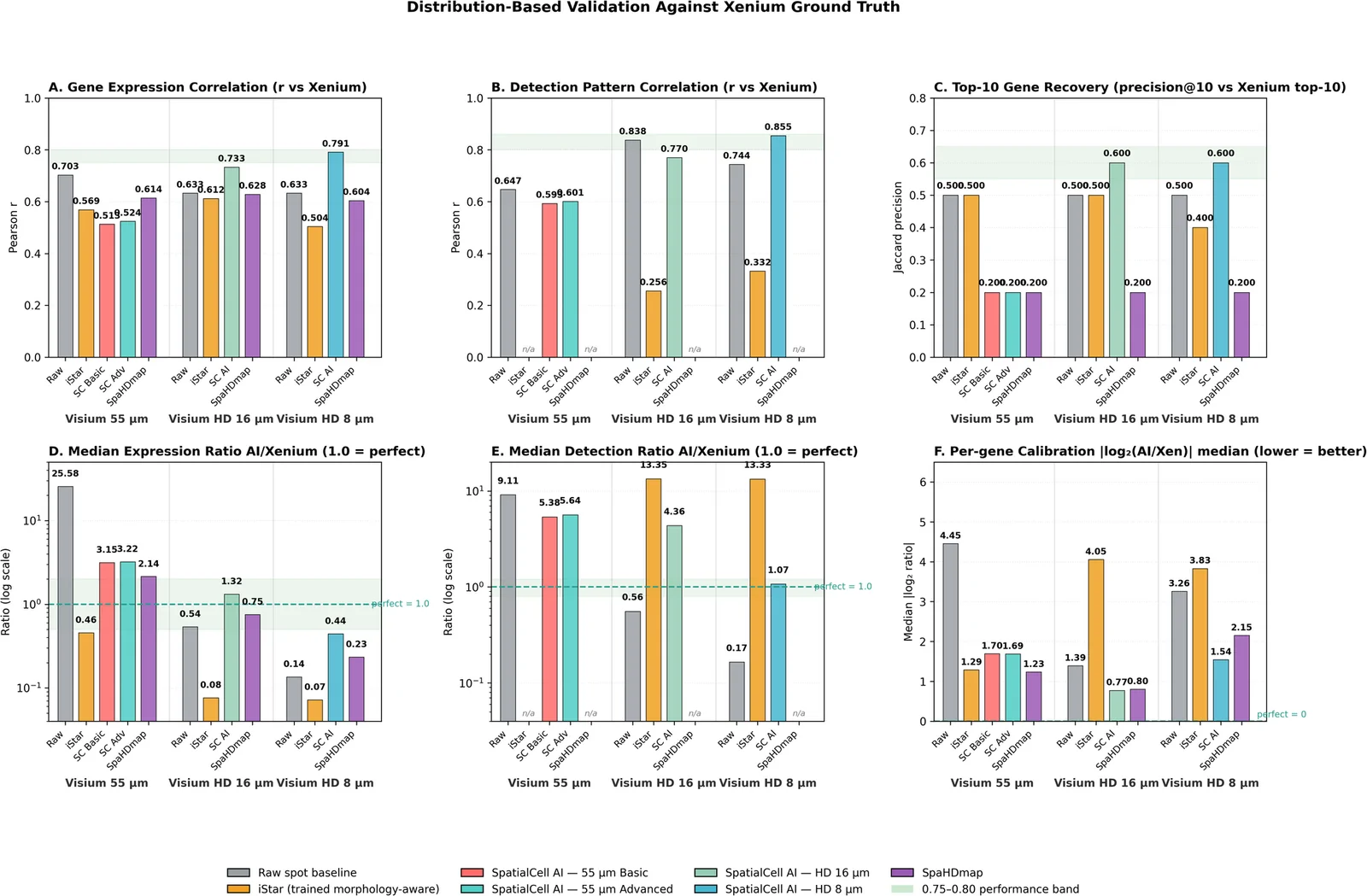
Distribution-Based Validation Across All Methods Against Xenium Ground Truth. Distribution-based validation across all methods on the matched Oliveira P2 colorectal cancer sample, against the same Xenium single-cell reference. Bars show full-sample values for raw and SpatialCell AI entries; matched-crop values for iStar and SpaHDmap. Bars grouped by input platform (Visium 55 µm, HD 16 µm, HD 8 µm). Genes used: 408 Visium ∩ Xenium panel for raw, iStar, and SpatialCell AI entries; panel ∩ HVG subset for SpaHDmap. Six panels: **(A)** Gene expression correlation r. **(B)** Detection pattern correlation r. **(C)** Top-10 gene recovery (precision@10). **(D)** Median expression ratio AI/Xenium (log y-axis; 1.0 = perfect; green band = within 2-fold). **(E)** Median detection ratio (log y-axis; 1.0 = perfect). **(F)** Per-gene calibration |log₂(AI/Xenium)| median (lower = better). Bar colours match Fig. 3.

To compare SpatialCell AI against the raw spot baselines and the trained morphology-guided methods iStar^17^ and SpaHDmap^18^, we evaluated all methods on six distribution-based metrics using the 408 Visium ∩ Xenium panel genes against the same Xenium single-cell ground truth, with SpaHDmap restricted to the panel ∩ HVG intersection per its default pipeline (Methods). Figure 3 shows the input-resolution trend for each metric, revealing how each method’s performance changes across platforms; Fig. 4 provides the detailed per-method values as bars grouped by input platform.

Gene expression correlation measures whether a method recovers the correct per-gene mean expression profile at the population level: for each gene, we compute the mean expression across all output units and correlate the resulting 408-gene vector against Xenium’s. On the Visium 55 μm input, the raw spot baseline leads (*r* = 0.703) because each spot already averages ~ 10–20 cells and provides a good population-level summary; SpatialCell AI (0.513 Basic, 0.524 Advanced), iStar (0.569), and SpaHDmap (0.614 on the panel ∩ HVG subset) score lower because redistributing a multi-cell mixture across individual cells introduces noise into the gene mean without adding information (Figs.  4A). The pattern reverses on HD inputs: at HD 16 μm, SpatialCell AI (0.733) overtakes the raw baseline (0.633), iStar (0.612), and SpaHDmap (0.628); at HD 8 μm, SpatialCell AI reaches the headline *r* = 0.791 — well inside the 0.75–0.80 performance band — while iStar (0.504) drops below the raw baseline (0.633) and SpaHDmap remains broadly flat at 0.604 (Figs.  4A). The matched-crop trend in Fig. 3A — and the equivalent matched-crop trends for the other five metrics in Fig. 3B–F — confirms the same pattern on a strict head-to-head comparison region; per-panel numerical values are in Supplementary Table S1. The architectural explanation is that as input bins approach single-cell scale, SpatialCell AI’s training-free integration aggregates fine-grained signals that genuinely improve over the raw bins, whereas trained per-gene super-resolution methods degrade because per-bin supervision becomes sparse and noisy^17, 15^.

Detection pattern correlation assesses whether a method preserves which genes are commonly detected and which are rare, by correlating the per-gene detection-rate vectors (fraction of units with expression > 0) between the method and Xenium. SpatialCell AI HD 8 μm achieves the highest detection correlation of any method (*r* = 0.855), overtaking the raw HD 8 μm baseline (0.744) (Figs. 3B and 4B). Among the raw baselines, HD 16 μm achieves the highest detection correlation (0.838) because 16 μm bins occupy a sweet spot: they are large enough (~ 2–5 cells per bin) to reliably detect expressed genes without excessive technical dropout, yet small enough that rarely-detected genes are not inflated to near-universal detection as they are in 55 μm spots (~ 15 cells per spot, detection *r* = 0.647); conversely, the finer 8 μm bins (~ 1 cell) suffer more technical dropout, reducing detection rates and adding noise (0.744). iStar’s Visium 55 μm entry is undefined because its regression-based output is continuous-valued with no exact zeros^17^, so the detection rate is identically 100% for every gene — an architectural property of regression models, not a defect. Where iStar’s detection correlation is defined (HD 16 μm: 0.256; HD 8 μm: 0.332), the values are substantially lower than both the raw baselines and SpatialCell AI, indicating that iStar does not faithfully preserve the sparsity structure of the gene panel (Figs. 3B and 4B). SpaHDmap’s detection-r is uninformative across all platforms because its continuous output is nonzero at essentially every pixel (Methods).

To test whether SpatialCell AI correctly identifies the most highly expressed genes — the marker-discovery use case — we computed the Jaccard overlap between each method’s top 10 most-expressed genes and Xenium’s top 10. Both SpatialCell AI HD variants tie at precision@10 = 0.60, the highest of any method, while raw baselines and iStar score 0.50 or below and SpaHDmap remains at 0.20 across all three platforms (Figs. 3C and 4C). The SpatialCell AI 55 μm variants score lower (0.20–0.30), consistent with the redistribution of multi-cell spot expression across individual cells on the 55 μm input.

Expression magnitude calibration quantifies how accurately a method recovers absolute expression levels, measured as the median ratio of the method’s per-gene mean to Xenium’s (Figs. 3D and 4D; log scale, 1.0 = perfect; green band marks the within-2-fold zone 0.5–2.0×). Notably, SpatialCell AI moves the expression ratio closer to the ideal 1.0 on every input platform: on 55 μm, the raw baseline over-estimates by 25.58× while SpatialCell AI reduces this to 3.15–3.22× (an 8-fold improvement in calibration, even though expression correlation is lower on this input) and SpaHDmap reaches 2.14×; on HD 16 μm, the raw baseline under-estimates at 0.54× while SpatialCell AI achieves 1.32× — the closest calibration of any method on any platform — and SpaHDmap reaches 0.75× (near-parity); on HD 8 μm, the raw baseline under-estimates at 0.14× while SpatialCell AI recovers to 0.44× and SpaHDmap drops to 0.23×. By contrast, iStar systematically compresses expression magnitudes to 0.07–0.46× across all three platforms, consistently under-estimating and never improving over the raw baseline on magnitude calibration — indicating that iStar’s per-gene neural network does not preserve absolute expression levels. Detection-rate calibration tells a parallel story (Figs. 3E and 4E): SpatialCell AI HD 8 μm achieves near-perfect detection parity with Xenium (ratio 1.07 — the only method inside the 0.8–1.2 near-perfect band), while the raw 55 μm baseline over-detects by 9.11× because multi-cell spots inflate detection rates. iStar’s HD outputs massively over-detect (13.33× on HD 8 μm, 13.35× on HD 16 μm) — worse even than the raw 55 μm spots — because iStar’s regression model produces near-universal nonzero predictions for most genes, inflating detection rates far above the true single-cell levels. iStar’s 55 μm detection ratio is undefined (all detection rates = 100%, as noted in Panel B). SpaHDmap’s detection ratio is uninformative for the same reason as Panel B.

Finally, the per-gene calibration |log₂(AI/Xenium)| median provides a symmetric measure of how tightly each method’s gene-level magnitudes track Xenium (Figs. 3F and 4F; lower = better). Unlike Panels D and E which use raw ratios, this metric penalises over- and under-estimation equally (2× over and 2× under both score 1.0). SpatialCell AI HD 16 μm achieves the best calibration of any method (median |log₂| = 0.77, meaning the typical gene is within 1.7-fold of Xenium), with SpaHDmap HD 16 μm a close second (0.80). SpaHDmap 55 μm (1.23), iStar 55 μm (1.29), and SpatialCell AI HD 8 μm (1.54) form the next tier; SpaHDmap HD 8 μm (2.15) sits below this. The raw 55 μm baseline is worst (4.45, reflecting ~ 22× count inflation), and iStar on the HD platforms performs poorly (4.05 on HD 16 μm, 3.83 on HD 8 μm — worse than every other method on those platforms).

Across the six distribution-based metrics, the SpatialCell AI HD variants win or tie for first place on four (expression r, detection r, precision@10, detection ratio) and achieve the best or near-best calibration on the remaining two (expression ratio, |log₂| calibration). No competing method wins on any of the six. The consistent pattern — visible as trends in Fig. 3 and as bars in Fig. 4 — is that SpatialCell AI’s training-free integration adds value above the raw baseline only on HD inputs where each bin is at or below cellular scale. On the 55 μm input, SpatialCell AI’s value is not metric improvement over raw spots but the production of individual cell records with spatial coordinates and per-cell expression — a qualitative output that raw spots cannot provide (Fig. 1).

### Robustness across two additional orthogonal metrics

To test whether SpatialCell AI’s advantage generalises beyond the distribution-based framework, we computed two additional orthogonal per-gene metrics: per-gene calibration log-ratio (a symmetric measure of magnitude bias) and per-gene Moran’s I correlation against Xenium^23^(a measure of spatial structure preservation). Per-method values for both metrics are shown in Supplementary Figure S2.

On per-gene calibration log-ratio (Supplementary Figure S2A), SpatialCell AI HD 16 μm achieves a median log₂ of − 0.04 — the only method whose median sits essentially at zero, reflecting near-perfect magnitude calibration. SpaHDmap on the panel ∩ HVG subset follows a platform-dependent pattern (median log₂ = +1.07 / −0.52 / −2.15 across 55 μm / HD 16 μm / HD 8 μm), reaching closest to zero on HD 16 μm. By contrast, iStar systematically deviates from Xenium by 1.18 to 4.05 log₂ units across platforms, and the raw 55 μm baseline deviates by 4.45 log₂ units.

On per-gene Moran’s I correlation against Xenium (Supplementary Figure S2B), SpatialCell AI HD 16 μm achieves the best non-raw value (0.781), exceeding SpaHDmap HD 16 μm (0.509) and substantially exceeding iStar HD 16 μm (0.435). At HD 8 μm, SpatialCell AI scores 0.447 — lower than the raw HD 8 μm bins (0.779) but above SpaHDmap (0.373) and two orders of magnitude above iStar HD 8 μm (0.007).

### Head-to-head benchmark against morphology-guided baselines

The broader deconvolution landscape has been surveyed across three benchmarking studies^12, 13, 24^ and is summarised in *Morphology-guided methods and the position of SpatialCell AI*. Among the recent morphology-guided methods (iStar, SpaHDmap, GHIST, Thor, PRTS; Table 1, see *Morphology-guided methods and the position of SpatialCell AI*), iStar^17^ and SpaHDmap^18^ were evaluated head-to-head against SpatialCell AI across all three Visium input platforms (55 μm, HD 16 μm, HD 8 μm) on matched crops of the Oliveira P2 sample. SpatialCell AI is the only method in this comparison that combines training-free, reference-free, and per-cell output (Table 1). Full procedural details (pretrained weights, panel ∩ HVG gene-set choice for SpaHDmap, and per-method scoring) are given in Methods. Per-method numbers are in Supplementary Table S1 and visualised in Fig. 3 (input-resolution trends) and Fig. 4 (bar chart).

#### The input-resolution architectural finding

The strict matched-crop comparison reveals an **opposite trend** (Fig. 3) for iStar and SpatialCell AI across the three Visium input platforms on the same biological sample. Every value is on the same physical region of tissue per platform, with the same 408 panel-overlap genes against the same Xenium reference. Two clean findings emerge:

1. **iStar provides essentially no improvement over the raw spot baseline on any Visium platform** when both are evaluated on the same crop. The deltas are + 0.011 / −0.002 / −0.020 across 55 μm / HD 16 μm / HD 8 μm — all within noise.

2. **SpatialCell AI provides a substantial improvement over the raw spot baseline on HD inputs** when both are evaluated on the same crop: +0.100 r-units on HD 16 μm and + 0.253 r-units on HD 8 μm. On Visium 55 μm SpatialCell AI is essentially tied with the raw baseline.

The crop region is representative of the full sample: SpatialCell AI’s HD 8 μm crop score (0.777) is within 0.014 r-units of the full-sample score (0.791).

The slope-plot visualisation in Fig. 3 makes the trends immediately visible: the SpatialCell AI line ascends, the iStar line descends and crosses SpatialCell AI at the HD 16 μm midpoint, and the SpaHDmap line remains broadly flat across the three platforms.

### Pseudo-spot deconvolution benchmark against reference-based methods

To benchmark against reference-based deconvolution methods, we aggregated Xenium CRC P2 cells into a 55 μm-pitch pseudo-spot grid^22^ (matching Visium spot geometry; 2,000 spots, median 29 cells per spot, 25 graph-cluster categories from the Xenium Ranger output as ground-truth labels) and ran Tangram and cell2location with their authors’ recommended training budgets. Tangram^25^ achieved a mean per-cluster Pearson of 0.759 (median 0.828), and cell2location achieved 0.760 (median 0.817) with tighter per-spot fraction estimates by MAE (0.030), RMSE (0.075), and Jensen–Shannon divergence (0.151). Per-method per-spot prediction tables are deposited in the GitHub repository (see Data availability).

Because these methods produce fundamentally different outputs — Tangram and cell2location predict per-spot cell-type fractions, while SpatialCell AI produces per-cell expression — each is evaluated on its own native metric (Supplementary Table S2 for deconvolvers; Supplementary Table S1 and Figs. 3 and 4 for SpatialCell AI).

### Sensitivity to histology perturbations

To characterise the segmentation step’s robustness, we performed controlled perturbation experiments on the Oliveira P2 H&E (10,000 × 10,000 px crop) under nine conditions: Gaussian noise (σ = 5, 10, 20), resolution downsampling (75%, 50%, 25%), and stain normalisation (Macenko, Vahadane) (Supplementary Table S3, Supplementary Figure S1).

Resolution downsampling has remarkably small effect on cell count: even at 25% resolution, 99.6% of baseline cells are recovered with mean displacement < 7 pixels. Gaussian noise causes graceful degradation up to a clear threshold — σ ≤ 5 is harmless, while σ = 20 loses ~ 52% of cells.

 By contrast, both Macenko^26^ and Vahadane stain normalisation reduce the segmented cell count by 75% and drop mask IoU to 0.03 (Supplementary Table S3).

## Discussion

### Consistency across validation metrics

The validation results in *Distribution-based validation across all methods* (Figs. 3 and 4) and *Robustness across two additional orthogonal metrics* (Supplementary Figure S2) together establish that SpatialCell AI’s advantage on the Oliveira P2 sample is robust across independent measurements. Across six distribution-based metrics in Figs. 3 and 4, and two additional orthogonal metrics in Supplementary Figure S2, the SpatialCell AI HD variants lead on the majority, with no competing method winning on any. This consistency across eight independent measurements provides strong evidence that SpatialCell AI’s HD outputs genuinely capture the biology that the Xenium reference encodes, rather than performing well on a single numerical aspect of it. The trade-off visible at HD 8 μm — Moran’s I lower than the raw bins (see *Robustness across two additional orthogonal metrics*) — reflects an expected reduction in per-gene spatial autocorrelation when reconstructing per-cell expression from fine-grained inputs, and is dwarfed by the gap to iStar at the same resolution.

The convergence of these eight independent metrics has direct implications for downstream biology. The HD 16 μm variant in particular satisfies three independent requirements for reliable per-cell analysis: (i) gene-level magnitudes match Xenium on the typical gene (calibration log₂ ≈ 0; |log₂| = 0.77), enabling quantitative comparison of expression levels between cell types; (ii) per-gene spatial autocorrelation patterns track Xenium with correlation 0.781 — the strongest of any non-raw method — supporting identification of tissue niches and cellular neighbourhoods; and (iii) the top 10 most-expressed genes overlap with Xenium’s at precision@10 = 0.60, the highest of any method, enabling marker-discovery analyses. Together, these properties establish SpatialCell AI HD 16 μm as the configuration in which the per-cell output is most reliable for the standard downstream applications of single-cell spatial analysis.

### The input-resolution architectural distinction

The head-to-head comparison against iStar and SpaHDmap (Fig. 3, Supplementary Table S1; numbers in The *input-resolution architectural finding*) reveals a clean architectural distinction: **training-free methods become advantageous on HD inputs**,** while trained methods track the raw baseline regardless of input resolution.**

The reason is architectural: iStar^17^ trains a per-gene neural network from each input bin as a supervision example, so as bins shrink from 55 μm to 8 μm and per-bin counts become sparser, the per-gene predictor fits a noisier signal and the predicted super-resolution grid degrades. SpatialCell AI’s training-free integration assigns expression to each segmented cell from nearby spots, so finer bins reduce mixing across cells and the integrated per-cell signal becomes cleaner. SpaHDmap^18^ fits a low-rank metagene model (rank = 20 by default) per dataset and projects per-pixel embeddings through that basis to recover per-gene maps; the compression smooths per-gene signal toward the metagene modes regardless of input resolution, consistent with its broadly flat trend across platforms. SpatialCell AI’s specific niche is therefore the HD-input regime, and the validation in this manuscript demonstrates that it occupies that niche cleanly.

The practical implication is that the choice of method depends on the input platform: for standard Visium 55 μm, SpatialCell AI’s value is the production of per-cell records with spatial coordinates — a qualitative output that raw spots cannot provide, even though the population-level gene-mean correlation does not improve. For Visium HD at 8–16 μm resolution, SpatialCell AI provides both per-cell output AND quantitative metric improvement over the raw data and over the competing morphology-guided methods.

SpatialCell AI is the first method to combine training-free operation, reference-free expression integration, and per-cell output granularity — a combination not shared by any prior method (Table 1). This means SpatialCell AI can be applied to any tissue, any patient, and any spot-based platform without requiring a matched scRNA-seq reference, per-dataset model training, or paired subcellular spatial data.

## Conclusion

SpatialCell AI is the first spatial transcriptomics framework to combine training-free operation, reference-free expression integration, and per-cell output granularity. On a matched colorectal cancer sample validated against Xenium single-cell ground truth, the SpatialCell AI HD variants achieve the highest expression correlation of any method tested (*r* = 0.791 on Visium HD 8 μm) and lead on the majority of six distribution-based validation metrics, with no competing method winning on any. The input-resolution trend reveals a clear architectural distinction: SpatialCell AI’s performance improves monotonically as input bins approach single-cell scale, while the trained morphology-guided methods iStar and SpaHDmap track the raw baseline on every platform — demonstrating that training-free integration is the right approach for the HD-input regime.

On Visium HD inputs (8 μm and 16 μm), SpatialCell AI delivers both per-cell resolution and substantial quantitative improvement over both the raw data and competing morphology-guided methods. On standard Visium 55 μm, SpatialCell AI transforms spot-level data into individual cell records with spatial coordinates and per-cell expression — an output that raw spots cannot provide and that opens the door to per-cell spatial analyses on the most widely used and affordable spatial transcriptomics platform. Cross-tissue generalisability, sensitivity to segmentation quality on diverse pathologies, and pipeline refinements for the HD 8 μm input regime remain to be evaluated in future work.

Reference-based deconvolution methods such as Tangram and cell2location (see *Pseudo-spot deconvolution benchmark against reference-based methods*) achieve strong performance on their native per-spot cell-type fraction task, but they require external scRNA-seq reference data, do not integrate tissue morphology, and produce per-spot fractions rather than per-cell expression profiles — limiting their applicability for downstream per-cell spatial analyses.

### Limitations

The quantitative validation in this manuscript uses one matched colorectal cancer sample (Oliveira et al. 2025, Patient 2). While the four pipeline variants span the range of spot-based input platforms and the validation includes six distribution-based metrics plus two additional orthogonal metrics, generalisation across tissues, patients, fixation protocols, and pathologies has not been evaluated and is a priority for future work.

Validation is restricted to the 408 genes shared between the Xenium 422-gene panel and the Visium / Visium HD transcriptome-wide assays. The remaining approximately 17,600 Visium genes cannot be evaluated against single-cell ground truth, and performance on genes outside the Xenium panel is therefore extrapolated.

The pipeline’s accuracy depends on the quality of the nuclear segmentation. The integration step is deterministic given a set of nuclear polygons; consequently, the entire pipeline inherits the performance characteristics of the underlying segmentation. The robustness experiments in *Sensitivity to histology perturbations* identify the practical operating envelope for H&E input, demonstrating compatibility with a wide range of H&E imaging conditions. The pipeline also supports immunofluorescent (IF) input (Methods Step 1); IF perturbation robustness has not been evaluated in this manuscript and is a priority for future work.

## Supplementary Information

Below is the link to the electronic supplementary material.

## Data Availability

All validation datasets generated in this study are available at https://zenodo.org/records/15794331. The complete validation framework and analysis code are available at https://github.com/Spatialcell/spatialcell-ai-validation/tree/main. Raw spatial transcriptomics data used for validation were obtained from https://www.10xgenomics.com/platforms/visium/product-family/dataset-human-crc. The SpatialCell AI platform for data processing is accessible at https://spatialcell.tech.
