# Supplementary material for: SpatialCell AI achieves reference-free single-cell resolution from spot-based spatial transcriptomics through morphology-guided enhancement
Source: Sci Rep. 2026 Jun 16;16:27636. doi: 10.1038/s41598-026-55246-w (PMC13538567; doi:10.1038/s41598-026-55246-w)
Supplement: Supplementary file 1 [file 41598_2026_55246_MOESM1_ESM.zip › supplementary material/Supplementary_Legends_ESM.docx]

# Supplementary Figure and Table Legends

**Manuscript:** SpatialCell AI achieves reference-free single-cell resolution from spot-based spatial transcriptomics through morphology-guided enhancement

**Author:** Abdalla Elbialy (aelbialy@spatialcell.tech)

**DOI:** 10.1038/s41598-026-55246-w

Legends for Supplementary Figures S1–S2 and Supplementary Tables S1–S3.

## Supplementary Figure S1 — Histology robustness

Nine controlled perturbations applied to a representative 10,000 × 10,000 px crop of the Oliveira P2 H&E, each re-segmented by the SpatialCell AI nuclear segmentation step. **Top row:** perturbed H&E images (1.5k × 1.5k px central crops). **Bottom row:** segmentation overlays (red = detected nucleus). Conditions: Gaussian noise (σ = 5, 10, 20), resolution downsampling (75%, 50%, 25%), and stain normalisation (Macenko, Vahadane). *Vahadane fell back to Macenko on the available analysis backend. Quantitative metrics (cell counts, mask IoU, centroid displacement) are reported in Supplementary Table S3 and discussed in the* Sensitivity to histology perturbations* section.

## Supplementary Figure S2 — Two orthogonal per-gene metrics across all methods

Two additional per-gene metrics on the same Oliveira P2 sample, grouped by input platform. **A. Per-gene calibration log-ratio distribution** — boxplot per method of log₂(AI mean / Xenium mean); the dashed green line at 0 marks perfect calibration. **B. Per-gene Moran’s I correlation vs Xenium** — bars show the per-gene Pearson correlation between the method’s per-gene Moran’s I vector and the Xenium reference Moran’s I vector, measuring how well each method preserves spatial autocorrelation across the gene panel. SpaHDmap entries are scored on the panel ∩ HVG subset; all other entries on the 408-gene Visium ∩ Xenium panel. Bar colours match Figure 3.

## Supplementary Table S1 — Head-to-head benchmark: all methods on the matched Oliveira P2 sample

Distribution-based validation metrics for all methods on the Oliveira P2 colorectal cancer sample using 408 Visium ∩ Xenium panel genes (or panel ∩ HVG intersection for SpaHDmap) against Xenium ground truth. Full-sample and crop-matched values are reported for each SpatialCell AI variant. Columns: Method, n units, n genes, Expression r (Pearson against Xenium per-gene means), Detection r (Pearson against Xenium per-gene detection rates), Expression ratio (median AI/Xenium per-gene mean). iStar’s super-resolution output for the 55 µm input is continuous-valued with no exact zeros; detection rate is identically 1.0 for every gene, so the correlation is undefined. SpaHDmap is scored on the panel ∩ HVG intersection per its default pipeline (246 / 215 / 212 genes across the three platforms; see Methods).

## Supplementary Table S2 — Pseudo-spot deconvolution benchmark

Per-cluster Pearson correlation between predicted and true cell-type fractions on 2,000 Xenium-derived pseudo-spots (25 clusters). Tangram and cell2location evaluated with recommended training budgets. Columns: Method, n spots, n clusters, Mean Pearson, Median Pearson, MAE, RMSE, Mean Jensen–Shannon divergence (JSD).

## Supplementary Table S3 — Histology perturbation robustness

Segmentation performance under nine controlled perturbations on a 10,000 × 10,000 px crop of the Oliveira P2 H&E. Mask IoU and centroid displacement measured against the unperturbed baseline. Columns: Condition, n cells, Mask IoU vs baseline, Mean displacement (px). Conditions include baseline, three Gaussian noise levels (σ = 5, 10, 20), three resolution downsampling levels (75%, 50%, 25%), and two stain normalisation methods (Macenko, Vahadane). Vahadane fell back to Macenko on the available analysis backend; both rows are reported for completeness.
