# Supplementary material for: SpatialCell AI achieves reference-free single-cell resolution from spot-based spatial transcriptomics through morphology-guided enhancement
Source: Sci Rep. 2026 Jun 16;16:27636. doi: 10.1038/s41598-026-55246-w (PMC13538567; doi:10.1038/s41598-026-55246-w)
Supplement: Supplementary file 1 [file 41598_2026_55246_MOESM1_ESM.zip › supplementary material/supplementary_figure_S2_extended_metrics.pdf]

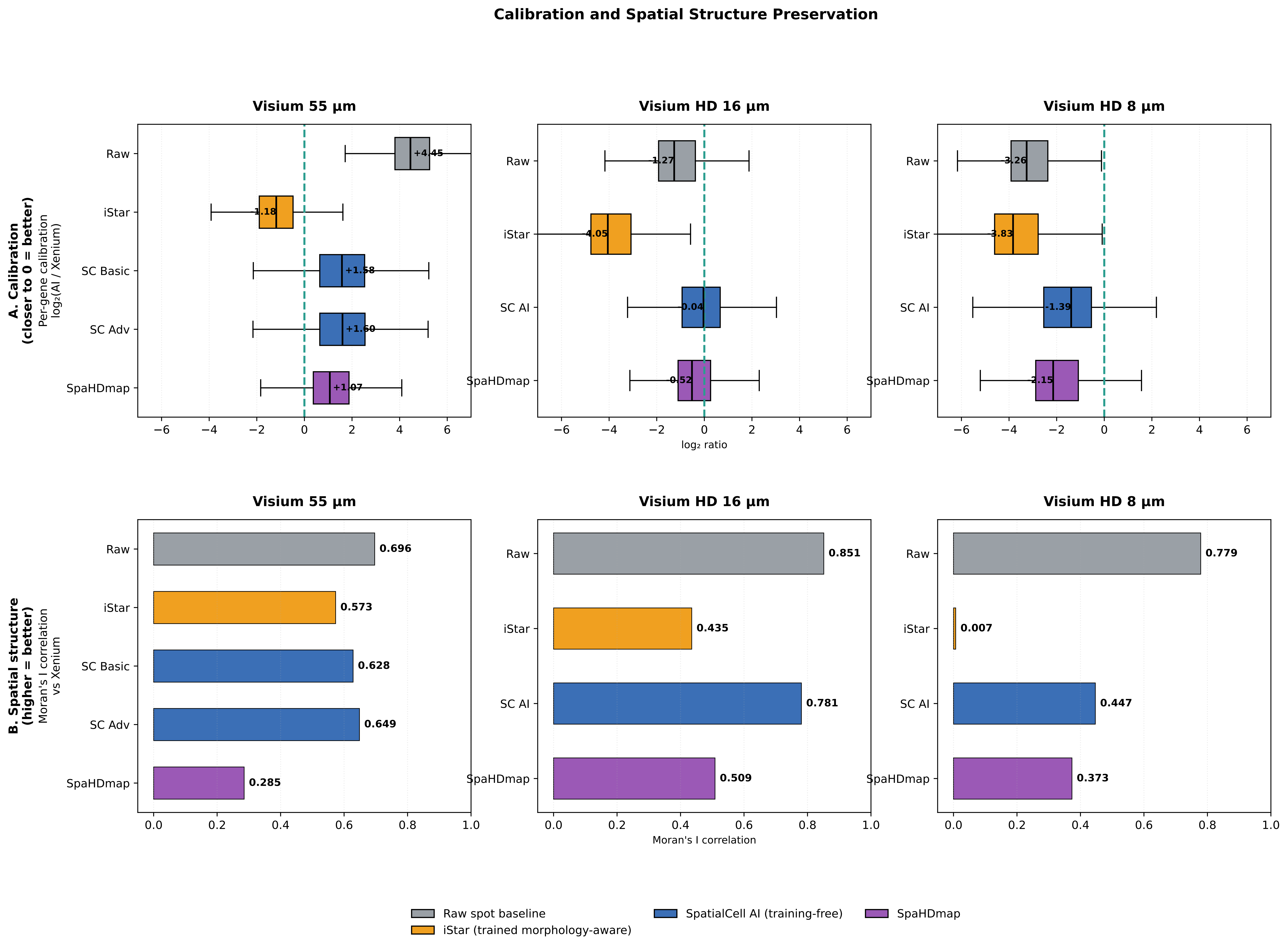

Calibration and Spatial Structure Preservation
A. Calibration
(closer to 0 = better)
Per-gene calibration
log₂(AI / Xenium)
Visium 55 µm
Visium HD 16 µm
Visium HD 8 µm
Raw
iStar
SC Basic
SC Adv
SC AI
SpaHDmap
+4.45
-1.18
+1.58
+1.60
+1.07
-1.27
-4.05
-0.04
-0.52
-3.26
-3.83
-1.39
-2.15
log₂ ratio
B. Spatial structure
(higher = better)
Moran's I correlation
vs Xenium
0.696
0.573
0.628
0.649
0.285
0.851
0.435
0.781
0.509
0.779
0.007
0.447
0.373
Moran's I correlation
Raw spot baseline
iStar (trained morphology-aware)
SpatialCell AI (training-free)
SpaHDmap
