# Supplementary material for: SpatialCell AI achieves reference-free single-cell resolution from spot-based spatial transcriptomics through morphology-guided enhancement
Source: Sci Rep. 2026 Jun 16;16:27636. doi: 10.1038/s41598-026-55246-w (PMC13538567; doi:10.1038/s41598-026-55246-w)

Histology Perturbation Robustness

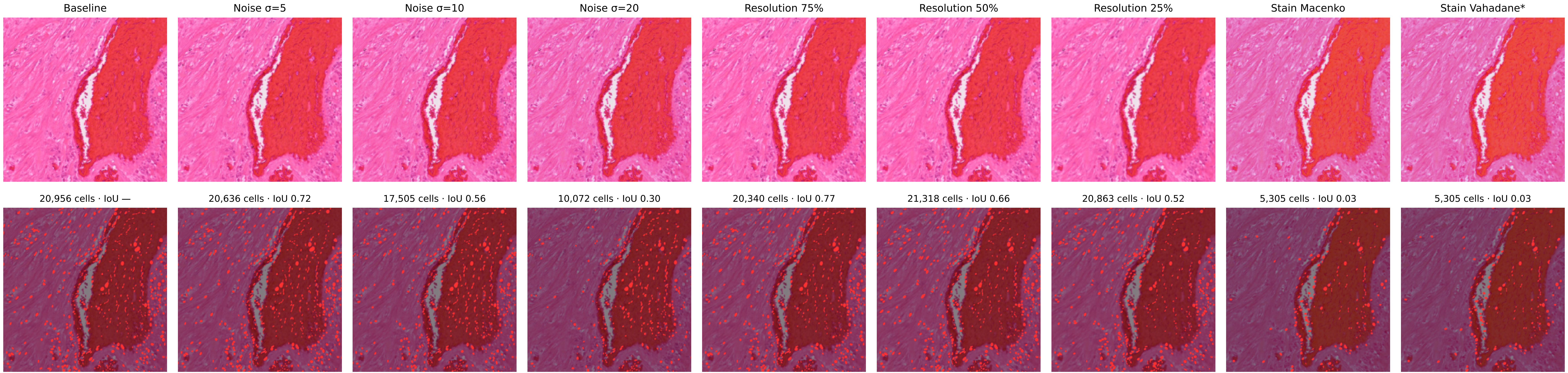

Supplement: Supplementary file 1 [file 41598_2026_55246_MOESM1_ESM.zip › supplementary material/supplementary_figure_S1_perturbations.pdf]
